# Case report: Cannabinoid therapy for discoid lupus erythematosus in a dog

**DOI:** 10.3389/fvets.2024.1309167

**Published:** 2024-02-09

**Authors:** Maria Eduarda Schmitz da Silva, Bruna Christianetti, Erik Amazonas, Marcy Lancia Pereira

**Affiliations:** ^1^Veterinary Medicine Student, Federal University of Santa Catarina (UFSC), Curitibanos, Brazil; ^2^Department of Biosciences and One Health, Center for Rural Sciences, Federal University of Santa Catarina (UFSC), Curitibanos, Brazil; ^3^Cannabis Development and Innovation Center (PODICAN/UFSC), Curitibanos, Brazil

**Keywords:** discoid lupus erythematosus, cannabinoid therapy, CBD, THC, autoimmune disease, cannabis

## Abstract

Discoid lupus erythematosus (DLE) is a common autoimmune skin disease in dogs. Conventional treatments, such as corticosteroids, can be effective but often have side effects. This case report presents a successful use of cannabinoid therapy (CT) in a dog with DLE resistant to conventional treatment. A 2-year-old mixed-breed dog with a history of DLE presented with worsening lesions despite treatment with corticosteroids and other medications. Liver enzymes levels were elevated, indicating corticosteroid-induced side effects. CT with a CBD-rich full spectrum Cannabis oil was initiated. The dosage was gradually adjusted until the minimum effective dose was found. Within a few weeks of starting CT, the dog showed significant improvement in skin lesions and in liver enzymes levels. After 1 year, the dog remains clinically stable on a low dose of full-spectrum CBD-rich oil. No evidence of DLE recurrence was observed. This case suggests that CT may be a viable alternative or complementary therapy for DLE in dogs, particularly for those experiencing adverse effects from conventional treatments. Further research is warranted to confirm the efficacy and safety of CT for DLE management in dogs.

## 1 Introduction

Discoid lupus erythematosus (DLE) is an immune-mediated skin disease that affects dogs of both sexes and breeds from the age of 2. It is caused by the production of antibodies against cellular components (autoantibodies) of the skin and leads to hypersensitivity reactions of type II or III (1). Autoantibodies target healthy skin cell components, particularly nuclear structures and ribosomal proteins, initiating the inflammatory cascade. Pro-inflammatory cytokines, like IL-6 and TNF-alpha, act as amplifiers, recruiting inflammatory cells and boosting their destructive tendencies, leading to tissue damage and visible lesions (2, 3). The Complement cascade also plays a crucial role in DLE as it perforates cell membranes, raising more tissue damage. As a result of the inflammatory cascade present on DLE, free radicals accumulate, amplify the inflammation, and wreak havoc by damaging cells and tissues.

The clinical signs of DLE include depigmentation, hair loss, and redness, which can progress to crusting and ulceration. The lesions are most common on the nose and ears, but they can also occur on the limbs, genitals, and mouth. The definitive diagnosis of DLE is made by physical examination, medical history, and histopathological examination (2, 4, 5).

Conventional immunosuppressive treatments such as corticosteroids (6) and calcineurin inhibitors (7) can be effective but often have side effects. Cannabinoids represent a novel class of immunomodulating compounds that are being thoroughly studied for diverse inflammatory and auto-immune diseases (8–13). These Cannabis-derived molecules act upon the endocannabinoid system (ECS) of vertebrate animals and utterly aims the maintenance of homeostasis throughout the intracellular environment across all body systems (14). While the exact mechanism of action for cannabinoids in DLE in dogs remains under investigation, their immunomodulatory effects through the endocannabinoid system (ECS) offer a promising explanation for their therapeutic potential. Cannabidiol (CBD) and Tetrahydrocannabinol (THC) inhibit mast cell degranulation, reducing the release of inflammatory mediators like histamine and prostaglandins (15). CBD and THC also downregulates the production of pro-inflammatory cytokines interleukin (IL)-6 and tumor necrosis factor (TNF)-alpha (15), while promoting the release of anti-inflammatory cytokines like IL-10 and promotes the activity of regulatory T cells (Tregs) (16), which suppress the overall immune response and prevent excessive inflammation and scavenge free radicals preventing oxidative stress (17). CBD can inhibit the activation of the Complement cascade, avoiding the recruitment of more inflammatory cells. Known for its analgesic and anti-pruritic effects, THC seems to act through activation of CB1 receptors in sensory neurons, which inhibits the transmission of itch signals to the spinal cord and brain, providing direct relief from scratching and discomfort. THC can suppress the release of the neuropeptide substance P, which contributes to neurogenic inflammation and itch sensation, and thus reduces neurogenic inflammation (18).

This case report presents the successful use of cannabinoid therapy (CT) in a dog with DLE resistant to conventional treatment.

## 2 Case description

A 2-year-old female mixed-breed dog weighing 25.5 kg and with a body condition score of 5 (on a scale of 1 to 9) was presented to the Veterinary School Clinic (CVE) of the Federal University of Santa Catarina with a previous histopathological diagnosis of discoid lupus erythematosus. The main complaint was epidermal scaling, depigmentation, and crust formation in the nasal bridge region and inside the nostrils. Previously, the dog had been treated with topical tacrolimus (Tacroz^®^ 1 mg, ointment, BID), vitamin E (DrogaVET^®^, 400 IU, 1 capsule PO, BID), and a sunscreen and hydration lotion (Hydra Reflex^®^ lotion, applied before sun exposure) for 30 days, but no improvement of the lesions was observed.

**Figure 1:**
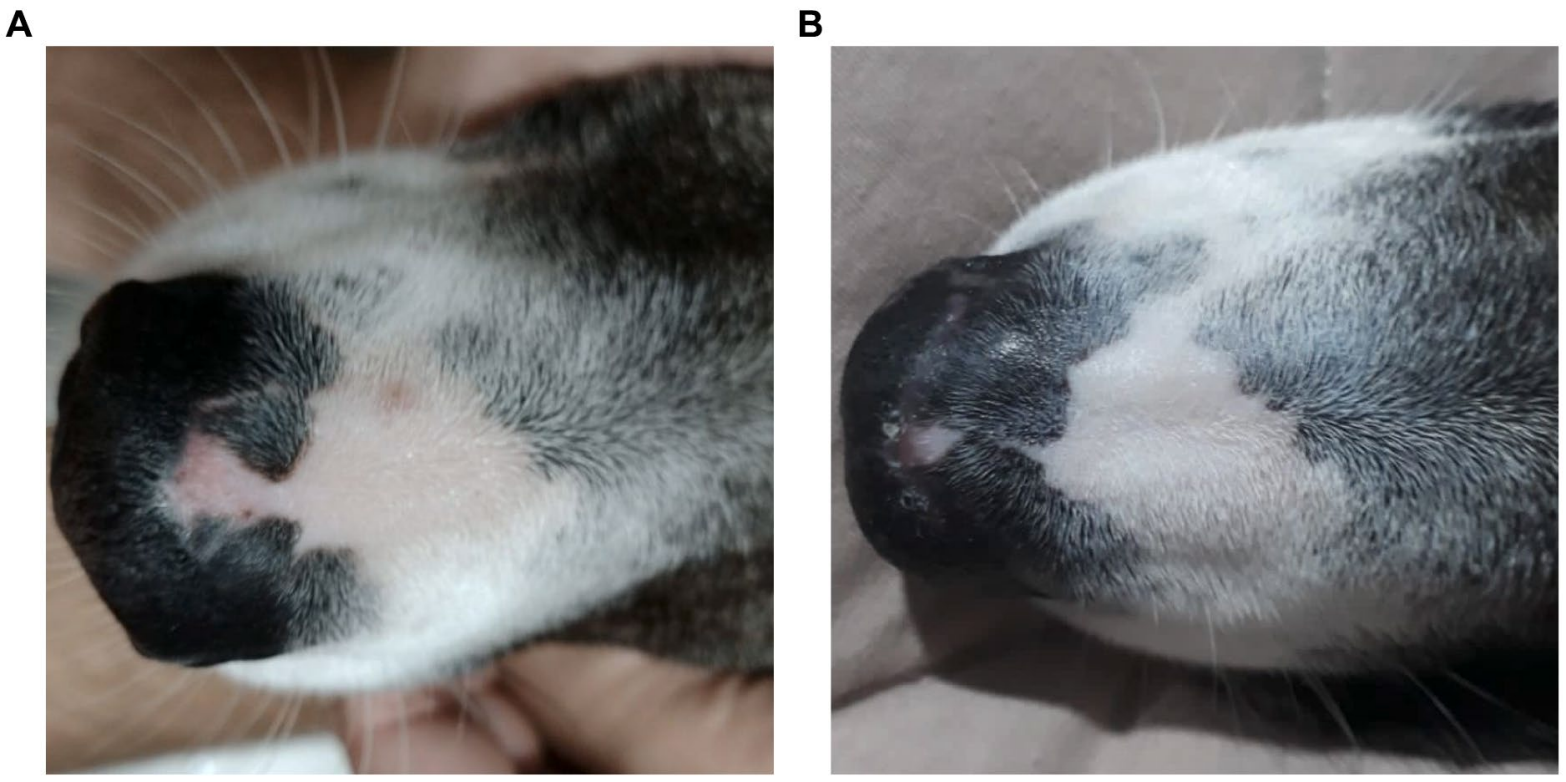
A female, mixed-breed dog, 2 years old, presented with epidermal depigmentation, on the nasal bridge. **(A)** Macroscopic image showing depigmentation in the nasal planum prior to treatment with Cannabis oil (date: 05/31/2022). **(B)** Macroscopic image showing the reduction and stabilization of depigmentation in the nasal planum after 1 year and 3 months of treatment with Cannabis oil (date: 09/19/2023).

Upon physical examination, the nasal region presented with 0.8 mm hypopigmented areas, diffuse erythema with erosion, and desquamation. Treatment with 1.5 mg/kg prednisolone (40 mg Eurofarma generic, 1 tablet PO, BID) was initiated for 2 weeks. While the dog exhibited slight improvement upon a one-month follow-up, the lesions persisted (Figure 1A). Corticosteroid therapy was extended for another 2 weeks, unfortunately leading to a worsening of the lesions.

During 7 months, three attempts to reduce the corticosteroid dosage proved unsuccessful. The dog developed behavioral changes, including increased irritability with its housemates, weight gain from 25.5 kg to 36.7 kg, a 44% increase in 7 months, and indications of liver damage. Liver function tests conducted 1 month apart confirmed these concerns, showing elevated levels of alanine aminotransferase (ALT) (160 U/L and 276.6 U/L respectively, reference range: 10–88 U/L) and alkaline phosphatase (FA) (181 U/L and 416 U/L respectively, reference range: 20–156 U/L).

No baseline tests were performed to measure the patient’s condition before initiating corticosteroid therapy. Prior to the initial examination, no complaints beyond peeling, depigmentation, and crusting were documented. The emergence of behavioral and weight concerns only occurred following the administration of corticosteroids.

With corticosteroid therapy no longer an option, the owner, concerned about the dog’s well-being, explored alternative treatments. After discussion, it was decided to interrupt corticosteroid therapy and to try cannabinoid therapy (CT) with cannabis derivatives.

The patient was directed to a veterinarian specialist well-versed in cannabinoid therapy. The veterinarian prescribed a full-spectrum oil containing a 2:1 THC:CBD ratio (20 mg/mL THC, 10 mg/mL CBD, and a total of 40 mg/mL, considering other non-identified cannabinoid species). All Cannabis-based products used along this dog’s treatment were provided by the following non-governmental medical Cannabis associations in Brazil: Cannabis Sem Fronteiras (CSF), AMA-ME, Alternativa, and Santa Cannabis, to whom we express our gratitude for their timely delivery and generous donation of several Cannabis oils throughout the treatment.

Cannabinoid treatment began with a single drop of the cannabis oil (0.08 mg/kg/day total cannabinoids; AMA-ME) administered orally once daily for 3 days. The dose was then gradually increased every 3 days, progressing from one oral drop once daily to one drop twice daily (0.16 mg/kg/day total cannabinoids), then to two drops twice daily (0.32 mg/kg/day total cannabinoids), and so on, until the optimal dose for symptom control was identified. Interestingly, the owner reported an improvement in the dog’s behavior shortly after discontinuing prednisolone and within the first day of receiving the cannabis oil.

Forty days later, the patient returned with ear discomfort. Cytology swabs revealed yeast fungal otitis in the left ear and bacterial otitis with cocci and rods in the right ear. An ear cleaner compound containing 50% panthenol, 20% glycyrrhizic acid, 20% Lactic Acid, 3% mint essential oil and 2% chamomile essential oil (Oto Clean Up^®^, one spray per ear, once daily for 3 days) and an anti-inflammatory, antibiotic and antifungal otological suspension (49% orbifloxacin, 5.14% mometasone furoate and 5.14% Posaconazole; Posatex^®^_,_ eight drops per ear, once daily for 6 days, starting after the initial cleaning) were prescribed. While the owner reported administering eight drops of the 2:1 THC:CBD oil (40 mg/mL) twice daily (1.28 mg/kg/day total cannabinoids; AMA-ME), no significant improvement in the skin condition was observed. To address this, the protocol was adjusted to include 10 drops of a full-spectrum CBD-rich oil (50 mg/mL) twice daily (1.96 mg/kg/day total cannabinoids; Alternativa) while reducing the THC-dominant oil (40 mg/mL) to three drops once daily (0.24 mg/kg/day total cannabinoids; AMA-ME). Within a few weeks, the dog exhibited significant improvement in dermatological signs, accompanied by a concurrent improvement in liver function.

Ten days after the previous evaluation the dog returned for a follow-up appointment. The nasal planum lesion continued to shrink, prompting an increase in the CBD-rich oil dose from 10 to 15 drops (2.4 mg/kg/day total cannabinoids; Alternativa). The THC-rich oil dosage remained unchanged at three drops (0.24 mg/kg/day total cannabinoids; CSF). While mild erythema, discharge, and hair loss persisted in the left ear, the prescribed ear solution was continued for another 3 days, leading to complete resolution of the bilateral otitis externa.

Fifteen days later, a follow-up assessment revealed no further reduction in the area of nasal planum depigmentation, though its progression had stabilized. The patient’s overall condition had demonstrably improved with weight loss, and the owner reported a return to normal, playful behavior, with no observable signs of discomfort. Both cannabis oils doses were maintained at the previous levels.

Approximately 1 month and a half after the previous consultation, a follow-up revealed the nasal planum lesion to be static, exhibiting no further improvement or deterioration. However, a new lesion characterized by depigmentation and signs of allergic conjunctivitis was identified on the medial aspect of the right nostril. Keravit^®^ eye ointment (topically, twice daily for 5 days) was prescribed. Cannabis therapy remained unaltered, and the patient was advised to minimize sun exposure.

Three months later, the animal returned with no sign of depigmentation on the nasal planum. However, new crusted lesions were observed on the vulva. The affected area was cleaned with chlorhexidine 1% (Asseptcare spray^®^, BID for 7 days), while the dosages of the cannabis oils were maintained. Although the possibility of a lupus-related lesion was mentioned, no further diagnostic investigation was pursued. A subsequent phone follow-up confirmed complete resolution of the vulvar lesion.

Two months later, the animal returned for a clinical reassessment and annual vaccination. Peripheral blood was collected for a comprehensive evaluation, including biochemical analysis, blood cell count, and an antinuclear antibody (ANA) test. Despite the ongoing treatment, the animal remained clinically stable. The blood count revealed a discrete erythrocytosis, with elevated red blood cell count (8.79 × 10^6^/μl; reference: 5.5–8 × 10^6^/μl), hemoglobin (20.8 d/dL; reference: 12–18 g/dL), and hematocrit (65.1%; reference: 37–55%). Additionally, anisocytosis, polychromasia, macroplatelets, lipemia and hemolysis were observed in the serum. Notably, ALT levels, while still exceeding the reference range, had decreased to 123.6 U/L (10–88 U/L). Encouragingly, the ANA test result was negative.

Given the ease of access and satisfactory results, the animal’s cannabinoid therapy transitioned to a single full-spectrum cannabis oil with a 3:1 CBD:THC ratio (40 mg/mL total cannabinoids, Alternativa). To determine the minimum effective dose, the owner was instructed to gradually taper the medication, reducing the cannabis oil by 1 drop (0.08 mg/kg total cannabinoids) every 3 days and monitoring for any regression in the treatment response. This titration schedule would continue until the optimal maintenance dose was established.

One-year post-diagnosis, the animal maintains clinical stability (Figure 1B) on a twice-daily cannabinoid dose of 0.32 mg/kg/day from Santa Cannabis at the same 3:1 CBD:THC ratio at 40 mg/mL, with a body weight of 26.6 kg, only 1 kilogram above initial measurement.

## 3 Discussion

**Figure 2:**
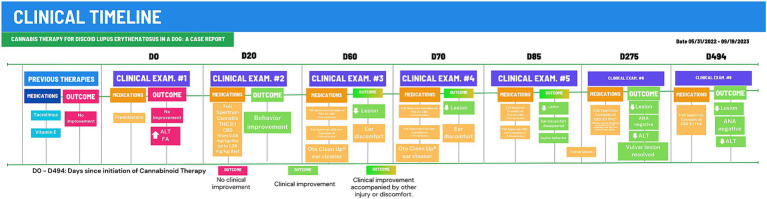
Clinical timeline for 494-days of Cannabinoid Therapy (CT). After 275 days of CT, the animal was stabilized and total cannabinoid dose was gradually reduced to 0.38 mg/kg/day. D0, First examination at CVE/UFSC; Outcomes in red boxes, no improvement or worsening; Outcomes um green boxes, clinical improvement.

Discoid lupus erythematosus (DLE) remains a mysterious foe in dogs. Its origins are shrouded in a mix of genetics, infections, hormones, and sun exposure (1). This autoimmune disease presents as scaly, discolored patches typically on the nose, but sometimes venturing to ears, lips, and beyond (2, 19). Diagnosis involves piecing together the clinical signs, skin tests, and bloodwork (19), though specific autoantibody tests often elude DLE’s grasp (4). Differential diagnoses include nasal pyoderma, demodicosis, dermatophytosis, erythematous or foliaceus pemphigus, dermatomyositis, uveodermatological syndrome, solar nasal dermatitis, and nasal depigmentation (2, 5, 20). Traditional therapies, like corticosteroids and calcineurin inhibitors, hold the fort initially, but often require long-term commitment due to DLE’s tendency to return, and carry potential side effects (2, 7). Weight gain and behavior changes were both side effects observed in the present case. The dog showed increased irritability with its housemates and the animal’s weight rose 44% from 25.5 kg to 36.7 kg during corticosteroid therapy. One month into cannabinoid treatment, the patient had already reduced weight reaching 32.4 kg (9% less) and ended up at 26.6 kg as of the final observation performed (Figure 2).

The patient’s initial presentation included persistent depigmentation and scaling on the nasal plan, along with crusted lesions on the ears and vulva. An episode of otitis externa was also reported. Increased ALT and FA enzymes were observed, relevant to the prolonged use of corticosteroids. However, no prior tests were performed before the start of corticosteroid treatment for comparison purposes. The animal was referred with a positive biopsy for DLE and a negative antinuclear antibody (ANA) test.

The treatment for DLE involves controlling the inflammatory pathways involved in the pathogenesis. Lesions typically respond well to a variety of medications. In most cases, treatment must be continued throughout the animal’s life due to frequent relapses after dose reduction, as observed in this case. In mild cases, DLE treatment consists of topical application of corticosteroids such as betamethasone, fluocinolone, or cyclosporine 1 to 2% twice daily (BID) until complete remission, which may take 4 to 6 weeks (3). The frequency of applications is then reduced to a minimum of every 24 or 48 h. Also, topical treatments with calcineurin inhibitors such as tacrolimus ointment 0.1% BID can be used in mild cases (2, 3, 21). Our canine patient, diagnosed with DLE, initially embarked on a journey of corticosteroids and tacrolimus ointment but this was ineffective in controlling the disease-derived lesions. Additionally, tapering proved treacherous, and the liver showed signs of distress. Vitamin E, a potential ally in symptom control, was unfortunately abandoned before its impact could be assessed. In more severe cases, immunosuppressive therapy is indicated. This may include systemic corticosteroids such as prednisone or prednisolone at a dose of 2 mg/kg SID or 1 mg/kg BID orally until lesions are healed, which may take up to 4 weeks, and there can be no improvement at all. Vitamin E or a combination of niacinamide and tetracycline as complementary treatments for the control of signs such as pruritus in DLE have been recommended (3). Cyclosporine is recommended for severe cases of DLE (20), but it is expensive and can be difficult to maintain treatment consistently. The major challenge lies in the search for an effective and safe long-term therapy.

Cannabis derivatives, beyond their pain-relieving prowess, are emerging as potential knights in shining armor against inflammation and immune system overwork in the veterinary world (8, 15, 22–26). Unlike traditional drugs, these compounds dance with the body’s own endocannabinoid system (ECS), a master conductor of cellular harmony, homeostasis and diverse functions (27). By influencing the immune response’s orchestra, they can silence the pro-inflammatory drums and amplify the anti-inflammatory melodies (15, 28–30). This intricate waltz aligns beautifully with DLE’s needs, as evidenced by the improvement seen in our patient.

Veterinarians are interested in the use of cannabinoid compounds derived from the *Cannabis sativa* plant as safe and effective alternatives for the treatment of DLE in dogs. Clinical studies and case reports in animals have shown positive results for the use of these compounds in the treatment of canine osteoarthritis (22–25, 31), epilepsy (31–34), behavioral disorders in dogs (35)and horses (36), and anti-inflammatory effects (37). However, no studies or reports have been conducted so far on the use of cannabis oil in the treatment of DLE in dogs.

As observed, conventional therapy with corticosteroids can be effective, but it is limited by its prolonged use. Cannabinoids, on the other hand, lack significant side effects and are safe for long-term usage (27, 31, 38, 39). Cannabinoid therapy for DLE, still in its infancy, needs meticulous adjustments and individualized doses. This case exemplifies the “Start low, go slow” mantra, where the total daily dose gently ascended from 0.08 mg/kg to 2.64 mg/kg, guided by the patient’s unique response. With each careful increase, the aim is to restore the ECS’s rhythm and find the perfect melody, the smallest effective dose, for each individual animal, acknowledging the diverse symphony of each ECS (40, 41).

Full-spectrum derivatives of *Cannabis sativa* contain hundreds of cannabinoids, such as THC and CBD, which act on various G protein-coupled receptors, including cannabinoid receptors 1 and 2 (CB1 and CB2), Transient receptor potential vanilloid (TRPV), Transient receptor potential melastatin (TRPM), Transient receptor potential ankyrin (TRPA) and the Peroxisome Proliferator-Activated Receptor (PPAR) (15, 27, 29). These receptors trigger myriad effects on cellular metabolism including important signaling pathways intrinsic to the immune response, such as AMPc, MAPK/ERK/MEK/FOS/JUN, and PI3/Akt (42) and modulate the cellular environment towards homeostasis and thus resolving inflammatory processes (15, 27, 29, 42).

Cannabinoids have been associated with the modulation of immune function and the inhibition of the release of pro-inflammatory cytokines, such as tumor necrosis factor alpha (TNF-α), interferon alpha, gamma, and beta (IFN-ɑ, IFN-ɣ, and IFN-β), interleukins IL-1β, IL-6, IL-12, IL-23, and regulates the nuclear factor NF-κB (15, 27, 43, 44). Cannabinoids also modulate the activation of T and B lymphocytes, promote the secretion of IL-10 and stimulate regulatory T cells, therefore reducing the inflammatory response (15, 29). CBD inhibits the release of IFN-ɑ selectively via CB2 in plasmacytoid dendritic cells, which are present in high levels in the skin of patients with discoid lupus erythematosus (DLE) (30). The hypersensitivity reactions that occur in DLE are responsible for pro-inflammatory mechanisms that lead to tissue infiltration and damage. Studies have shown that cannabinoids are able to inhibit these processes (45–47). This may explain the improvement seen in the patient in question.

Our yearlong DLE case highlights successful dose titration for an Individualized Cannabinoid Therapy (ICT) approach. The treatment initiated with a daily cannabinoid dose of 0.08 mg/kg. Notable clinical improvements on the nasal planum started to be observed at 0.32 mg/kg/day, prompting a further increase to 2.64 mg/kg/day. Following clinical stabilization, the dose was gradually reduced, achieving the minimum effective dose of 0.32 mg/kg/day total cannabinoids.

Throughout the treatment period, the dog exhibited robust overall well-being, maintained an active and playful disposition, and experienced a stabilization of its dermatological signs. No corticoids were needed during the ICT. This offers initial indications that cannabinoids could potentially serve as a viable and health-conscious alternative to extended therapeutic approaches for DLE in dogs. The quest for definitive answers however continues: rigorous studies are needed to solidify the effectiveness of cannabis derivatives for DLE; the optimal dosage and administration schedule remain a melody waiting to be composed; and long-term safety and efficacy data require further research.

While the song of cannabis therapy for DLE in dogs holds immense promise, we must continue listening closely, gathering more evidence, and refining the tune. This case report adds its verse to the growing chorus, paving the way for future research and potentially offering a new rhythm of hope and a haven from the long-term reign of corticosteroids for dogs battling this challenging disease.

## Data availability statement

The raw data supporting the conclusions of this article will be made available by the authors, without undue reservation.

## Ethics statement

Ethical approval was not required for the studies involving animals in accordance with the local legislation and institutional requirements because this is a case report. The dog was ongoing clinical treatment at our university and we hereby describe the positive outcome of the cannabis treatment performed. Written informed consent was obtained from the owners for the participation of their animals in this study.

## Author contributions

MS: Data curation, Formal analysis, Investigation, Writing – original draft, Writing – review & editing. BC: Data curation, Formal analysis, Investigation, Writing – original draft, Writing – review & editing. EA: Conceptualization, Data curation, Investigation, Supervision, Validation, Writing – review & editing, Resources, Writing – original draft. MP: Conceptualization, Investigation, Supervision, Validation, Writing – review & editing, Data curation, Formal analysis, Project administration.

## References

[1] Nastri Gouvêa F Santos Pennacchi C Vieira Fernandes Ferreira A de Almeida P Luna Alves APP. Discoid lupus erythematosus in dog. Veterinária Notícias. (2022) 28:1–4. doi: 10.14393/VTN-v28n1-2022-62645

[2] Olivry T Linder KE Banovic F. Cutaneous lupus erythematosus in dogs: a comprehensive review. BMC Vet Res. (2018) 14:132. doi: 10.1186/s12917-018-1446-8, PMID: 29669547 PMC5907183

[3] Kurtdede A Alic Ural D. Guzel Ondokuz Mayıs Üniversitesi M. Cutaneous lupus erythematosus in a dog. Available at: https://www.researchgate.net/publication/287763990

[4] Pereira P Cristina Barini Nunes A Alves J Cunha DO Acb N Medvep PJ. Lúpus eritematoso discoide (LED)-Relato de caso em um canino SRD Discoid lupus erythematosus-Case report in a dog SRD. Alergologia Veterinária. (2014) 3:390–3.

[5] Banovic F Linder KE Uri M Rossi MA Olivry T. Clinical and microscopic features of generalized discoid lupus erythematosus in dogs (10 cases). Vet Dermatol. (2016) 27:488–e131. doi: 10.1111/vde.12389, PMID: 27747960

[6] Griffin CE Stannard AA Ihrke PJ Ardans AA Cello RM Bjorling DR. Canine discoid lupus erythematosus. Vet Immunol Immunopathol. (1979) 1:79–87. doi: 10.1016/0165-2427(79)90009-6 15612271

[7] Banovic F Robson D Linek M Olivry T. Therapeutic effectiveness of calcineurin inhibitors in canine vesicular cutaneous lupus erythematosus. Vet Dermatol. (2017) 28:493–e115. doi: 10.1111/vde.12448, PMID: 28439997

[8] Nagarkatti P Pandey R Rieder SA Hegde VL Nagarkatti M. Cannabinoids as novel anti-inflammatory drugs. Future Med Chem. (2009) 1:1333–49. doi: 10.4155/fmc.09.93, PMID: 20191092 PMC2828614

[9] Croxford JL Yamamura T. Cannabinoids and the immune system: potential for the treatment of inflammatory diseases? J Neuroimmunol. (2005) 166:3–18. doi: 10.1016/j.jneuroim.2005.04.023 16023222

[10] Shao K Stewart C Grant-Kels JM. Cannabis and the skin. Clin Dermatol. (2021) 39:784–95. doi: 10.1016/j.clindermatol.2021.05.006 34785006

[11] Banach D Ferrero P. Cannabis and pathologies in dogs and cats: first survey of phytocannabinoid use in veterinary medicine in Argentina. J Cannabis Res. (2023) 5:39. doi: 10.1186/s42238-023-00209-5, PMID: 38031164 PMC10685507

[12] Lima MG Tardelli VS Brietzke E Fidalgo TM. Cannabis and inflammatory mediators. Eur Addict Res. (2021) 27:16–24. doi: 10.1159/000508840 32726782

[13] Atalay S Jarocka-karpowicz I Skrzydlewskas E. Antioxidative and anti-inflammatory properties of cannabidiol. Antioxidants. (2020) 9:21. doi: 10.3390/antiox9010021, PMID: 31881765 PMC7023045

[14] Cital S Kramer K Hughston L Gaynor JS eds. Cannabis therapy in veterinary medicine. Cham: Springer International Publishing (2021).

[15] Anil SM Peeri H Koltai H. Medical Cannabis activity against inflammation: active compounds and modes of action. Front Pharmacol. (2022) 13:908198. doi: 10.3389/fphar.2022.908198, PMID: 35614947 PMC9124761

[16] Holloman BL Nagarkatti M Nagarkatti P. Epigenetic regulation of cannabinoid-mediated attenuation of inflammation and its impact on the use of cannabinoids to treat autoimmune diseases. Int J Mol Sci. (2021) 22:7302. doi: 10.3390/ijms22147302, PMID: 34298921 PMC8307988

[17] Khaksar S Bigdeli M Samiee A Shirazi-zand Z. Antioxidant and anti-apoptotic effects of cannabidiol in model of ischemic stroke in rats. Brain Res Bull. (2022) 180:118–30. doi: 10.1016/j.brainresbull.2022.01.001, PMID: 35031355

[18] Henry RJ Kerr DM Finn DP Roche M. For whom the endocannabinoid tolls: modulation of innate immune function and implications for psychiatric disorders. Prog Neuro-Psychopharmacol Biol Psychiatry. (2016) 64:167–80. doi: 10.1016/j.pnpbp.2015.03.006, PMID: 25794989

[19] Ferreira TC Pinheiro ADN Leite AKRM Guedes RFM Pinheiro DCSN. Pathogenesis, biomarkers and immunotherapy in autoimmune skin diseases in dogs and cats. A review. Rev Bras de Saude e Prod Anim. (2015) 9:299–319. doi: 10.5935/1981-2965.20150030

[20] Ataide W Silva V Ferraz H Amaral A Romani A. Lúpus Eritematoso Discoide Em Cães. Encicl Biosf. (2019) 16:995–1009. doi: 10.18677/encibio_2019a81

[21] Messinger L Strauss T Jonas L (2017). A randomized, double-blinded placebo controlled crossover study evaluating 0.03% tacrolimus ointment monotherapy in the treatment of discoid lupus erythematosus in dogs. Available at: www.symbiosisonline.orgwww.symbiosisonlinepublishing.com

[22] Gamble LJ Boesch JM Frye CW Schwark WS Mann S Wolfe L. Pharmacokinetics, safety, and clinical efficacy of Cannabidiol treatment in osteoarthritic dogs. Front Vet Sci. (2018) 5:165. doi: 10.3389/fvets.2018.00165, PMID: 30083539 PMC6065210

[23] Brioschi FA di Cesare F Gioeni D Rabbogliatti V Ferrari F D'Urso ES. Oral transmucosal cannabidiol oil formulation as part of a multimodal analgesic regimen: effects on pain relief and quality of life improvement in dogs affected by spontaneous osteoarthritis. Animals. (2020) 10:1–14. doi: 10.3390/ani10091505, PMID: 32858828 PMC7552307

[24] Verrico CD Wesson S Konduri V Hofferek CJ Vazquez-Perez J Blair E. A randomized, double-blind, placebo-controlled study of daily cannabidiol for the treatment of canine osteoarthritis pain. Pain. (2020) 161:2191–202. doi: 10.1097/j.pain.0000000000001896, PMID: 32345916 PMC7584779

[25] Mejia S Duerr FM Griffenhagen G McGrath S. Evaluation of the effect of Cannabidiol on naturally occurring osteoarthritis-associated pain: a pilot study in dogs. J Am Anim Hosp Assoc. (2021) 57:81–90. doi: 10.5326/JAAHA-MS-7119, PMID: 33450016

[26] Rieder SA Chauhan A Singh U Nagarkatti M Nagarkatti P. Cannabinoid-induced apoptosis in immune cells as a pathway to immunosuppression. Immunobiology. (2010) 215:598–605. doi: 10.1016/j.imbio.2009.04.001, PMID: 19457575 PMC3005548

[27] Copas G Amazonas E Brandon S. The pharmacology of cannabinoids In: Cital S Kramer K Hughston L Gaynor JS, editors. Cannabis therapy in veterinary medicine. Cham: Springer International Publishing (2021). 17–59.

[28] Robaina Cabrera CL Keir-Rudman S Horniman N Clarkson N Page C. The anti-inflammatory effects of cannabidiol and cannabigerol alone, and in combination. Pulm Pharmacol Ther. (2021) 69:102047. doi: 10.1016/j.pupt.2021.102047, PMID: 34082108

[29] de Lorimier L-P Hazzah T Amazonas E Cital S. Cannabinoids in oncology and immune response In: Cital S Kramer K Hughston L Gaynor JS, editors. Cannabis therapy in veterinary medicine. Cham: Springer International Publishing (2021). 231–69.

[30] Luz-Veiga M Azevedo-Silva J Fernandes JC. Beyond pain relief: a review on Cannabidiol potential in medical therapies. Pharmaceuticals. (2023) 16:155. doi: 10.3390/ph16020155, PMID: 37259306 PMC9958812

[31] Alvarenga IC Panickar KS Hess H Mcgrath S. Scientific validation of Cannabidiol for Management of dog and cat diseases. Annu Rev Anim Biosci. (2023) 11:227–46. doi: 10.1146/annurev-animal-081122 36790884

[32] McGrath S Bartner LR Rao S Packer RA Gustafson DL. Randomized blinded controlled clinical trial to assess the effect of oral cannabidiol administration in addition to conventional antiepileptic treatment on seizure frequency in dogs with intractable idiopathic epilepsy. J Am Vet Med Assoc. (2019) 254:1301–8. doi: 10.2460/javma.254.11.1301, PMID: 31067185

[33] Potschka H Bhatti SFM Tipold A McGrath S. Cannabidiol in canine epilepsy. Vet J. (2022) 290:105913. doi: 10.1016/j.tvjl.2022.105913, PMID: 36209995

[34] De Risio L Stabile F São Braz B Garcia GA. Safety and efficacy of cannabidiol-cannabidiolic acid rich hemp extract in the treatment of refractory epileptic seizures in dogs. Available at: https://www.epidiolex.com/sites/default/files/pdfs/VV-MED- 10.3389/fvets.2022.939966 PMC9372618 35967998

[35] Corsetti S Borruso S Malandrucco L Spallucci V Maragliano L Perino R. *Cannabis sativa* L. may reduce aggressive behaviour towards humans in shelter dogs. Sci Rep. (2021) 11:2773. doi: 10.1038/s41598-021-82439-2, PMID: 33531559 PMC7854708

[36] Cunha RZ Felisardo LL Salamanca G Marchioni GG Neto OI Chiocchetti R. The use of cannabidiol as a novel treatment for oral stereotypic behaviour (crib-biting) in a horse. Vet Anim Sci. (2023) 19:100289. doi: 10.1016/j.vas.2023.100289, PMID: 36824298 PMC9941357

[37] Gugliandolo E Licata P Peritore AF Siracusa R D’amico R Cordaro M. Effect of cannabidiol (Cbd) on canine inflammatory response: an ex vivo study on lps stimulated whole blood. Vet Sci. (2021) 8:185. doi: 10.3390/vetsci8090185, PMID: 34564578 PMC8473042

[38] de Andrade DF Gewehr JLH de Almeida EA. Safety and efficacy of the therapeutic use of Cannabis-based products in the treatment of dogs: an integrative review. Cannabis Cannabinoid Res. (2022) 7:736–44. doi: 10.1089/can.2021.0172, PMID: 35512739

[39] Lima TDM Santiago NR Alves ECR Chaves DSDA Visacri MB. Use of cannabis in the treatment of animals: a systematic review of randomized clinical trials. Anim Health Res Rev. (2022) 23:25–38. doi: 10.1017/S1466252321000189, PMID: 35703023

[40] MacCallum CA Russo EB. Practical considerations in medical cannabis administration and dosing. Eur J Intern Med. (2018) 49:12–9. doi: 10.1016/j.ejim.2018.01.004, PMID: 29307505

[41] Sideris A Lauzadis J Kaczocha M. The basic science of cannabinoids. Anesth Analg. (2024) 138:42–53. doi: 10.1213/ANE.0000000000006472, PMID: 38100799 PMC10788142

[42] López-Cardona AP Sánchez-Calabuig MJ Beltran-Breña P Agirregoitia N Rizos D Agirregoitia E. Exocannabinoids effect on in vitro bovine oocyte maturation via activation of AKT and ERK1/2. Reproduction. (2016) 152:603–12. doi: 10.1530/REP-16-0199, PMID: 27798282

[43] Shrivastava A Kuzontkoski PM Groopman JE Prasad A. Cannabidiol induces programmed cell death in breast cancer cells by coordinating the cross-talk between apoptosis and autophagy. Mol Cancer Ther. (2011) 10:1161–72. doi: 10.1158/1535-7163.MCT-10-1100, PMID: 21566064

[44] Preet A Qamri Z Nasser MW Prasad A Shilo K Zou X. Cannabinoid receptors, CB1 and CB2, as novel targets for inhibition of non-small cell lung cancer growth and metastasis. Cancer Prev Res. (2011) 4:65–75. doi: 10.1158/1940-6207.CAPR-10-0181, PMID: 21097714 PMC3025486

[45] Preteroti M Wilson ET Eidelman DH Baglole CJ. Modulation of pulmonary immune function by inhaled cannabis products and consequences for lung disease. Respir Res. (2023) 24:95. doi: 10.1186/s12931-023-02399-1, PMID: 36978106 PMC10043545

[46] Nagarkatti P Miranda K Nagarkatti M. Use of cannabinoids to treat acute respiratory distress syndrome and cytokine storm associated with coronavirus disease-2019. Front Pharmacol. (2020) 11:589438. doi: 10.3389/fphar.2020.589438, PMID: 33240092 PMC7677512

[47] Maayah ZH Ferdaoussi M Alam A Takahara S Silver H Soni S. Cannabidiol suppresses cytokine storm and protects against cardiac and renal injury associated with Sepsis. Cannabis Cannabinoid Res. (2022). doi: 10.1089/can.2022.0170, PMID: 36594988

