# Supplementary material for: Case report: Cannabinoid therapy for discoid lupus erythematosus in a dog
Source: Front Vet Sci. 2024 Feb 9;11:1309167. doi: 10.3389/fvets.2024.1309167 (PMC10884172; doi:10.3389/fvets.2024.1309167)
Supplement: Supplementary file 1 [file Data_Sheet_1.PDF]

CARE
case report guidelines

CARE Checklist of information to include when writing a case report

(cc) BY-NC-ND

Topic	Item	Checklist item description	Reported on Line
**Title**	1	The diagnosis or intervention of primary focus followed by the words “case report”	yes, line 01
**Key Words**	2	2 to 5 key words that identify diagnoses or interventions in this case report, including "case report"	line 11
**Abstract (no references)**	3a	Introduction: What is unique about this case and what does it add to the scientific literature?	line 16
	3b	Main symptoms and/or important clinical findings	lines 16-18
	3c	The main diagnoses, therapeutic interventions, and outcomes	lines 16-18
	3d	Conclusion—What is the main “take-away” lesson(s) from this case?	lines 16-18
**Introduction**	4	One or two paragraphs summarizing why this case is unique (**may include references**)	lines 33-42
**Patient Information**	5a	De-identified patient specific information.	lines 30
	5b	Primary concerns and symptoms of the patient.	lines 30-32
	5c	Medical, family, and psycho-social history including relevant genetic information	lines 33-37
	5d	Relevant past interventions with outcomes	lines 33-37
**Clinical Findings**	6	Describe significant physical examination (PE) and important clinical findings.	lines 53-57
**Timeline**	7	Historical and current information from this episode of care organized as a timeline	line 134
**Diagnostic Assessment**	8a	Diagnostic testing (such as PE, laboratory testing, imaging, surveys).	lines 46-52; 109-115
	8b	Diagnostic challenges (such as access to testing, financial, or cultural)	lines 59-71
	8c	Diagnosis (including other diagnoses considered)	line 48
	8d	Prognosis (such as staging in oncology) where applicable	lines 229-236
**Therapeutic Intervention**	9a	Types of therapeutic intervention (such as pharmacologic, surgical, preventive, self-care)	lines 55; 67
	9b	Administration of therapeutic intervention (such as dosage, strength, duration)	lines 72; 79; 82; 83; 85; 89-90; 102; 105; 117; 122.
	9c	Changes in therapeutic intervention (with rationale)	lines 72; 82; 83; 85; 89-90; 117; 122.
**Follow-up and Outcomes**	10a	Clinician and patient-assessed outcomes (if available)	lines 76; 82; 91; 96; 122
	10b	Important follow-up diagnostic and other test results	lines 78; 86; 94; 99; 105; 109-115
	10c	Intervention adherence and tolerability (How was this assessed?)	lines 76; 86
	10d	Adverse and unanticipated events	lines 76-77; 86
**Discussion**	11a	A scientific discussion of the strengths AND limitations associated with this case report	lines 178 - 228; 237-240
	11b	Discussion of the relevant medical literature **with references**.	lines 125-240
	11c	The scientific rationale for any conclusions (including assessment of possible causes)	lines 125-240
	11d	The primary “take-away” lessons of this case report (without references) in a one paragraph conclusion	lines 229-236
**Patient Perspective**	12	The patient should share their perspective in one to two paragraphs on the treatment(s) they received	lines 76-77; 81-83; 96-97
**Informed Consent**	13	Did the patient give informed consent? Please provide if requested	**Yes** [X] **No** [ ]
